# Expression of *Shigella flexneri gluQ-rs* gene is linked to *dksA* and controlled by a transcriptional terminator

**DOI:** 10.1186/1471-2180-12-226

**Published:** 2012-10-05

**Authors:** Valeria C Caballero, Viviana P Toledo, Cristian Maturana, Carolyn R Fisher, Shelley M Payne, Juan Carlos Salazar

**Affiliations:** 1 Program of Microbiology and Mycology, Institute of Biomedical Science (ICBM), Faculty of Medicine, University of Chile, Santiago, Chile; 2 Area of Biochemistry, Faculty of Dentistry, University of Chile, Santiago, Chile; 3 Molecular Genetics and Microbiology, The University of Texas at Austin, Austin, TX, USA; 4 Present address: Department of Molecular Genetics and Microbiology, Faculty of Biological Science, Catholic University, Santiago, Chile

**Keywords:** tRNA modification, Gene expression, Stringent response, Osmotic stress

## Abstract

**Background:**

Glutamyl queuosine-tRNA^Asp^ synthetase (GluQ-RS) is a paralog of the catalytic domain of glutamyl-tRNA synthetase and catalyzes the formation of glutamyl-queuosine on the wobble position of tRNA^Asp^. Here we analyze the transcription of its gene in *Shigella flexneri*, where it is found downstream of *dksA,* which encodes a transcriptional regulator involved in stress responses.

**Results:**

The genomic organization, *dksA-gluQ-rs*, is conserved in more than 40 bacterial species. RT-PCR assays show co-transcription of both genes without a significant change in transcript levels during growth of *S. flexneri*. However, mRNA levels of the intergenic region changed during growth, increasing at stationary phase, indicating an additional level of control over the expression of *gluQ-rs* gene. Transcriptional fusions with *lacZ* as a reporter gene only produced β-galactosidase activity when the constructs included the *dksA* promoter, indicating that *gluQ-rs* do not have a separate promoter. Using bioinformatics, we identified a putative transcriptional terminator between *dksA* and *gluQ-rs*. Deletion or alteration of the predicted terminator resulted in increased expression of the *lacZ* reporter compared with cells containing the wild type terminator sequence. Analysis of the phenotype of a *gluQ-rs* mutant suggested that it may play a role in some stress responses, since growth of the mutant was impaired in the presence of osmolytes.

**Conclusions:**

The results presented here, show that the expression of *gluQ-rs* depends on the *dksA* promoter, and strongly suggest the presence and the functionality of a transcriptional terminator regulating its expression. Also, the results indicate a link between glutamyl-queuosine synthesis and stress response in *Shigella flexneri*.

## Background

The fidelity of the translation process depends on the aminoacyl–tRNA synthetase enzymes (aaRS). These essential enzymes are responsible for the correct attachment of the corresponding amino acid onto the cognate tRNA, therefore organisms have at least 20 synthetases [1]. The enzymes are divided in two classes, each class having a conserved structure. The genes encoding the aaRS are easily detected within sequenced genomes [2,3], and some species contain synthetase gene duplications, such as the glutamyl-tRNA synthetases (GluRS) in *Acidithiobacillus ferrooxidans* and *Helicobacter pylori* (genes *gltX1* and *gltX2*) [4,5]. aaRS paralogs, predicted sequences with homology to fragments of synthetases, have also been identified, which is not surprising given the modular nature of the aaRS [6]. Some of the paralogs may be pseudogenes while others have known functions. For instance HisZ from *Lactococcus lactis*, which has similarity with the catalytic domain of histidyl-tRNA synthetase, is involved in histidine biosynthesis [7]. A recent study in *Salmonella enterica* has shown that PoxA, encoded by *poxA*/*genX*, has similarity to the carboxy-terminal catalytic domain of lysine-tRNA synthetase and is required for posttranslational aminoacylation of bacterial elongation factor P. A *poxA* mutant has reduced colonization and virulence, possibly due to misregulated expression of proteins encoded by the SPI-1 pathogenicity island [8,9].

An *Escherichia coli* glutamyl-tRNA synthetase paralog, glutamyl queuosine-tRNA^Asp^ synthetase (GluQ-RS) has approximately 35% amino acid similarity with the catalytic domain of GluRS. This includes the amino acids involved in recognition and activation of glutamate. Although GluQ-RS is missing the carboxyl-terminus domain responsible for the tRNA recognition, in *E. coli* this enzyme is able to activate the amino acid in the absence of the tRNA. Further, once the aminoacyl-adenylate has been formed, the enzyme attaches the glutamate to the nucleoside queuosine present onto the tRNA^Asp^. Therefore, this enzyme is involved in the synthesis of a new modified nucleoside glutamyl-queuosine (GluQ) present in tRNA^Asp^[10,11]. This modification is present in tRNA isolated under acidic conditions from bacterial cells grown in rich media. However, the enzyme is not essential for growth of *E. coli* in rich or minimal media [10]. Queuosine is widely distributed in bacteria, and it is present in the first base of the anticodon of tRNA^Asp^, tRNA^Asn^, tRNA^His^ and tRNA^Tyr^[12]; however in *E. coli* only tRNA^Asp^ is a substrate for the GluQ-RS enzyme.

The presence of modifications within the anticodon loop of the tRNA, could enhance the accuracy of the codon binding [13]. Then the tRNA^AspQ34^ might improve recognition of both GAC and GAU codons [14] and stimulate the binding of the GAU codon to the ribosome [15]. In *Shigella flexneri* it has been shown that mutations in genes required for tRNA modifications, *miaA* and *tgt* decreased virulence. *miaA* is required for 2-methylthio-N^6^-isopentenyladenosine modification at position 37 of the anticodon loop and *tgt* is involved in queuosine modification at position 34 within the anticodon loop [16-18]. In this study, we determined the role of the genome organization and its effect on the expression of the *gluQ-rs* gene in the major human pathogen, *S. flexneri.*

## Results

### Genomic organization of the *S. flexneri gluQ-rs* gene

**Figure 1:**
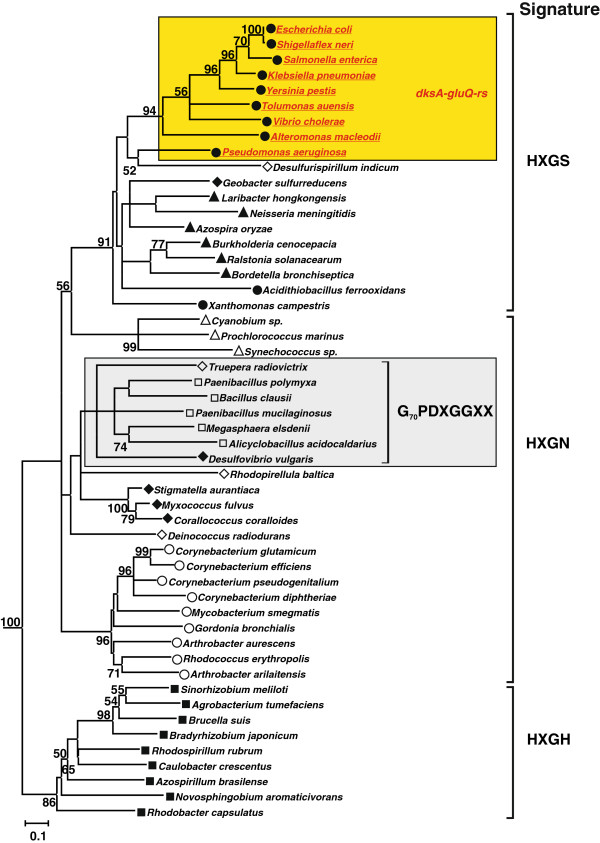
**GluQ-RS is distributed within the bacterial domain.** Rooted Phylogenetic analysis of selected sequences of GluQ-RS, showing the presence of this enzyme in the bacterial domain. Searching within the Uniprot database (http://www.uniprot.org/) of homologues protein to GluQ-RS were identified and then were searched in the GenomeNet (http://www.genome.jp/) database for confirmation and analysis of the genomic organization. Bootstrap values (>50%) where calculated by 400 replicates using the maximum-likelihood methods in the MEGA5 software [21] and rooted with archaeal GluRS from *Methanocaldococcus jannaschii* and *Archaeoglobus fulgidus* (not shown). In yellow background are shown bacterial species (in red and underlined) that are representatives having the genomic organization of *dksA-gluQ-rs* genes. The signature of each subgroup identified previously [11] is indicated. Filled symbols representing proteobacteria groups, open symbols represent other bacterial groups. ■: alphaproteobacteria, ▴: betaproteobacteria, : gammaproteobacteria, ♦: deltaproteobacteria, ○: actinobacteria, ▽: cyanobacteria, □: firmicutes, ◊: others.

GluQ-RS is required for the synthesis of the modified nucleoside, GluQ, present on tRNA^Asp^[10,11]. By searching the bacterial protein database Uniprot (http://www.uniprot.org/), we were able to identify GluQ-RS in more than a hundred bacterial species, primarily proteobacteria (Figure 1, filled symbols). From the phylogenetic analysis we can distinguished the three subgroups of enzymes described by Dubois et al., 2004 [11], which are characterized by the presence of the signature HXGS, HXGN or HXGH in the adenylate binding site. A similar tree was obtained using the Neighbor joining method. Phylogenetic analysis within the subgroup of enzymes with the HXGN motif, included representatives from the Firmicutes bacterial group (Figure 1, open square) together with *Desulfovibrio vulgaris* and *Truepera radiovictrix* enzymes. From the alignment, these members have 8 characteristic amino acids, G_70_PDXGGXX, that do not align with the other GluQ-RS (Figure 1, numbering is derived from *D. vulgaris* enzyme). Further genomic analysis indicated that the *gluQ-rs* gene is found primarily in two genomic arrangements, either alone or located immediately downstream of *dksA*. Searching within the String database [19] and GenomeNet [20], we found that the *dksAgluQ-rs* gene organization was conserved in more than 40 different species, all of which were within the gammaproteobacteria group. These included species of *Aeromonadales*, *Alteromonadales, Enterobacteriaceae*, including *E. coli* and *S. flexneri*, *Pseudomanadales*, and *Vibrionaceae* (Figure 1).

**Figure 2:**
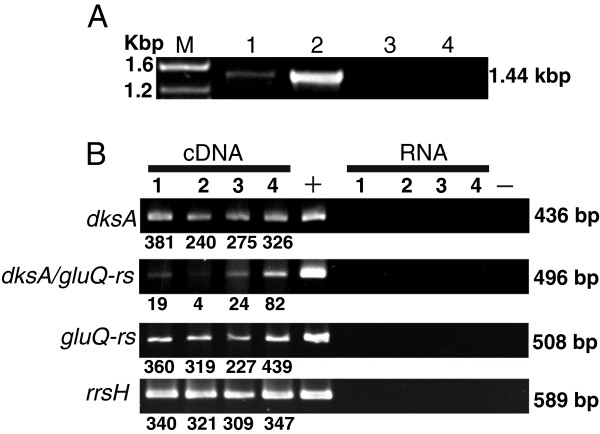
***gluQ-rs*is co-transcribed with*dksA*in*S. flexneri*2457T. A**) Agarose gel showing the amplified product of the full-length operon extending from the *dksA* gene through the end of *gluQ-rs* (1.44 kpb). Total RNA isolated during mid log phase growth of *S. flexneri* was subjected to reverse transcriptase and PCR (RT-PCR) using primers opeF/opeR (Table 2). **M**: molecular marker, sizes are indicated. **Lane 1**: RNA treated with reverse transcriptase, **Lane 2**: genomic DNA isolated from *S. flexneri* 2457T, **Lane 3**: RNA without reverse transcriptase treatment, **Lane 4**: negative control of PCR reaction without DNA. **B**) Electrophoretic analysis of each amplified gene fragment, *dksA* (dksAF/dksAR; 436 bp), *gluQ-rs* (gQRSF/gQRSR; 508 bp), the intergenic region *dksA/gluQ-rs* (interF/interR; 496 bp) and the ribosomal RNA 16S (*rrsH*F/*rrsH*R, 589 bp). Total RNA isolated during different phases of the growth curve of *S. flexneri* 2457T was subjected to RT-PCR to detect the corresponding fragment. **Lane 1**: lag phase, **Lane 2**: early mid log phase, **Lane 3**: mid log phase, **Lane 4**: stationary phase. **+**: corresponds to amplification using genomic DNA. **RNA**: Isolated RNA without reverse transcriptase treatment. **-**: negative control PCR reaction without DNA. Each band was estimated using Image J software (V1.46) and its amount was estimated using the fragment corresponding to 500 bp of the DNA ladder.

A bioinformatics analysis of the intergenic region between *dksA* and *gluQ-rs* showed great variation in the distance between the two genes among these bacterial species. In *S. flexneri* the intergenic region between the stop codon of *dksA* and the first codon of *gluQ-rs* is only 39 base pairs. Therefore, we suspected that the transcription of *gluQ-rs* was regulated by the previously characterized *dksA* promoter [22]. To test this hypothesis, we isolated total mRNA and performed RT-PCR to identify an mRNA that included both genes (Figure 2A). The observation that there is an mRNA species containing both genes (Figure 2A, lane 1) indicates that they are co-transcribed and that the expression of *gluQ-rs* may be regulated by the *dksA* promoter.

### *S. flexneri gluQ-rs* gene is co-transcribed with *dksA* gene

**Figure 3:**
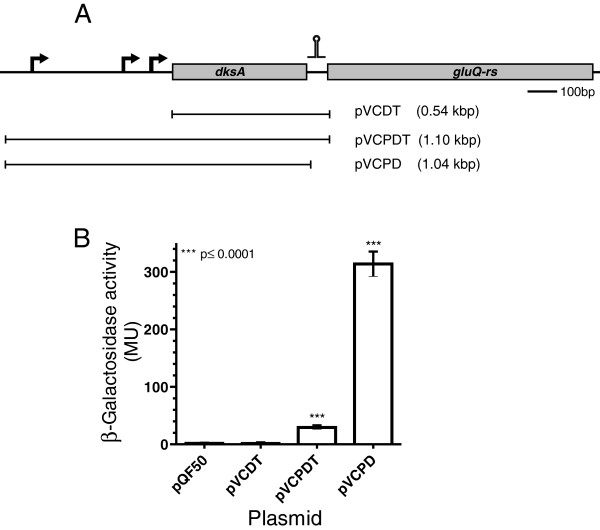
**The transcription of the*gluQ-rs*gene is controlled by a termination stem loop.A**) Schematic representation of the operon, the arrows indicate the position of each promoter identified by our bioinformatics analysis and experimentally determined by Kang and Craig, 1990 [22]. The putative ρ-independent terminator is represented by the stem loop symbol upstream of gluQ-rs gene. The horizontal bar represents the DNA region amplified and cloned into pQF50 (Table 1). The recombinant plasmids are described in Table 1. **pVCDT** does not have the *dksA* promoter but has the terminator. **pVCPDT** has the promoter region of *dksA* and the terminator upstream of *gluQ-rs*; therefore, it represents the genomic organization of the operon. **pVCPD** also has the promoter of *dksA* but lacks the terminator region. The size of each fragment is indicated. **B**) β-galactosidase activity of each protein extract obtained from the corresponding clone. The data represent the average of three experiments in triplicates and the Student *t* test was used to compare the means between each clone with the empty vector. *** *p* values <0.05 were considered statistically significant.

Although *S. flexneri gluQ-rs* can be transcribed from the *dksA* promoter, this did not rule out the presence of an additional, independent promoter. Therefore, the expression of each gene was measured by RT-PCR during different stages of *S. flexneri* growth in Luria Bertani (LB) at pH 7.4. The analysis of the *dksA* and *gluQ-rs* transcripts shows that for both mRNAs, the level is stable during the growth curve, with an increase of 1.3-fold at stationary phase compared to the early mid log phase (Figure 2B, compare lanes 2 and 4). However, the mRNA that includes the intergenic region showed variation depending on the stage of growth, increasing 20-fold at stationary phase compared with its expression at early mid log phase (Figure 2B*dksA/gluQ-rs*, compare lanes 2 and 4). In order to confirm those results, a transcriptional fusion strategy was used. Different segments of the operon were cloned and fused to the *lacZ* reporter gene in pQF50, and promoter activity was assayed by β-galactosidase activity [23]. Kang and Craig, 1990 [22] identified three promoters for *dksA*. By mean of bioinformatics tools, including BPROM from the Softberry software package (http://linux1.softberry.com/berry.phtml), we identified those promoters in *S. flexneri* and included all three promoters in the constructs indicated in Figure 3A. The plasmid containing a fragment from the *dksA* promoters to the beginning of the *gluQ-rs* gene, with the first five amino acids of GluQ-RS, named pVCPDT, represents the full length *dksA* gene with its native promoters (Table 1 and Figure 3A). A second fusion construct, pVCDT, contains sequence from the beginning of the coding region of *dksA* through the beginning of *gluQ-rs* and also included the first five amino acids of GluQ-RS. Because pVCDT does not have the *dksA* promoter region, it served as the reporter for transcription from an independent *gluQ-rs* promoter. A third construct, pVCPD, contained the segment from the *dksA* promoter to the end of the *dksA* gene, hence this plasmid does not have the intergenic region, nor the first amino acids of GluQ-RS (Table 1). Each of the recombinant plasmids was transformed into *S. flexneri* and the β-galactosidase activity was measured when the bacterial cells reached mid-log phase. Analysis of the enzymatic activity of these reporter fusions showed that the strain carrying pVCDT had baseline levels of the enzyme (Figure 3B), indicating that there is not an independent promoter for *gluQ-rs.* Thus, the promoter upstream of *dksA* is responsible for the expression of both genes, at least under the conditions assayed (see lane pVCPDT Figure 3B). Therefore, these two results (Figure 2 and Figure 3B) indicate that *dksA* and *gluQ-rs* form an operon, and *gluQ-rs* lacks an additional, separate promoter. A surprising observation was obtained with the clone containing pVCPD, which showed a ten-fold increase in enzymatic activity compared to pVCPDT (Figure 3B). This suggested the presence of a terminator or other regulatory sequence in the intergenic region that modulated the expression of *gluQ-rs*.

**Table 1:** Bacterial strains and plasmids used in this work

**Bacterial strains or plasmid**	**Characteristics**	**Source or reference**
*Shigella flexneri*		
*S. flexneri* 2457T	Wild type strain	Laboratory stock
*S. flexneri* 2457T Δ*gluQ-rs*::*kan*	Deletion mutant of *gluQ-rs* gene	This work
*Escherichia coli*		
*E. coli* W3110 Δ*gluQ-rs*::*kan*	Deletion mutant of *gluQ-rs* gene	[10]
DH5α	*F*^*-*^*ϕ80lacZΔM15 Δ(lacZYA-argF) U169 recA1 endA1 hsdR17 (rK-, mK+) phoA supE44 λ-thi-1 gyrA relA1*	[24]
BL21(DE3)	*F*^*-*^*ompT gal dcm lon hsdS*_*B*_*(r*_*B*_^*-*^*m*_*B*_^*-*^*) λ(DE3 [lacI lacUV5-T7 gene 1 ind1 sam7 nin5])*	Invitrogen
Plasmids		
pTZ57R/T	*bla*, pMB1 ori, *lacZ* peptide, f1 phage ori	Fermentas®
pQF50	*bla,* pMB1 ori, *lacZ* gene without promoter	[23]
pET15c	Empty vector, a modified version of pET15b	This work
pVCDT	*S. flexneri* fragment from nucleotide +58^a^ of *dksA* gene to beginning of *gluQ-rs* gene (+590) cloned into pQF50. Pair of primers used were PgluQF/PdksARCT.	This work
pVCPDT	*S. flexneri* fragment from nucleotide −506 of *dksA* gene to beginning of *gluQ-rs* gene (+590) cloned into pQF50. Pair of primers used were PdksAF/PdksARCT.	This work
pVCPDTMut	*S. flexneri* fragment from nucleotide −506 of *dksA* gene to beginning of *gluQ-rs* gene (+590) cloned into pQF50, with the terminator mutated by the nucleotides indicated in Figure 4a.	This work
pVCPD	*S. flexneri* fragment from nucleotide −506 of *dksA* gene to nucleotide +527 (end of *dksA* gene) cloned into pQF50. Pair of primers used were PdksAF/PdksARST.	This work
pATGGQRS	*S. flexneri* gene from nucleotide +509 (stop codon of *dksA*) to nucleotide +1469 (last codon of *gluQ-rs* without stop codon). Pair of primers used were ATGGQRSF/ATGGQRSR.	This work

^a^Fragments cloned are numbered based on the transcription start of *dksA* identified by [25].

### The *S. flexneri gluQ-rs* gene has an upstream transcription terminator

**Figure 4:**
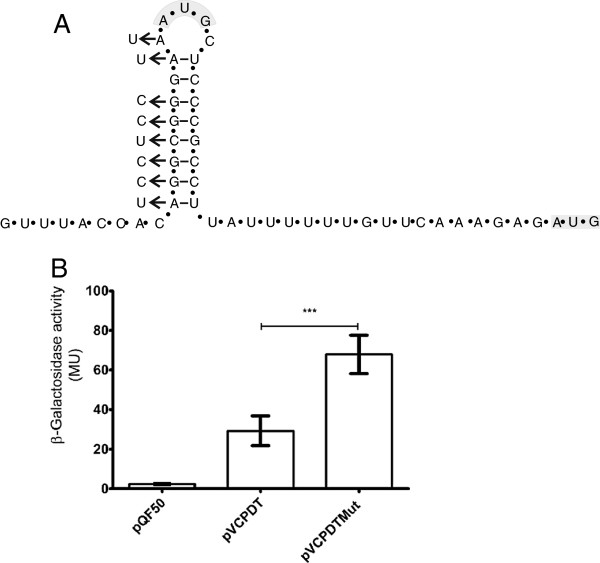
**Functionality of the transcriptional terminator upstream of*gluQ-rs*. A**) Schematic representation of the terminator with a ΔG = −14.7 Kcal/mol identified using Mfold software [26]. Bases shaded in grey indicate the two possible AUG start codons, one located in the bulge of the terminator structure and the other located 27 nucleotides downstream. The arrows indicate the site directed mutagenesis location, with the corresponding nucleotide changes designed to disrupt the predicted structure. **B**) β-galactosidase activity of protein extracts obtained from the corresponding clones. The plasmid **pVCPDTMut** has a similar construction as **pVCPDT** but contains the mutated terminator indicated above. The data represent the average of three experiments, each done in triplicate, and the Student *t* test was used to compare means between the pVCPDT and pVCPDTMut clones. *** *p* values <0.05 were considered statistically significant.

In order to explain the difference observed in expression of *lacZ* from the recombinant plasmids pVCPDT and pVCPD a bioinformatic analysis using mFold [26] was performed to search for possible secondary structures in the mRNA. A potential transcriptional terminator was found at the beginning of the *gluQ-rs* gene, leaving the first predicted AUG codon located on the bulge of this terminator (Figure 4A). In order to determine the functionality of this terminator, we performed site directed mutagenesis to disrupt the structure in the predicted stem (Figure 4A). As shown in Figure 4B, the plasmid containing the mutations, pVCPDTMut had >2-fold higher enzymatic activity (*p* < 0.05) than the plasmid containing the wild type sequence. This result suggested that the intergenic region upstream of *gluQ-rs* contains a transcriptional terminator.

### Identification of the first methionine

**Figure 5:**
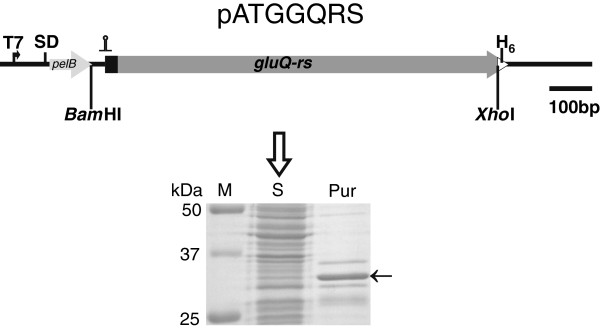
**Determination of the first methionine of GluQ-RS.** The cloning strategy utilized is shown at the top. A fragment from the stop codon of *dksA* to the end of *gluQ-rs* gene was amplified from *S. flexneri* genomic DNA with the primers ATGGQRSF/ATGGQRSR and cloned into a pET15c vector using the restriction sites *Bam*HI and *Xho*I. Therefore this clone represents the operon, but *dksA* was replaced with the plasmid encoded fragment *pelB*. The transcription of this plasmid, named pATGGQRS, is controlled by the T7 promoter and translation is controlled by the Shine-Dalgarno (SD) sequence of *pelB,* both contained within the plasmid. The GluQ-RS protein synthesized has a histidine tag (H_6_) at the C-terminus, facilitating protein purification. The putative ρ-independent terminator is represented by the stem loop symbol upstream of gluQ-rs gene, and the black box in the gene indicates the two possible peptides, depending of which methionine is utilized. Bottom: The SDS –polacrylamide gel electrophoresis showing the supernatant extract (S) and the partially purified protein (Pur) produced by cells carrying the recombinant plasmid. The predominant band indicated by the arrow was excised and subjected to amino terminal sequencing, yielding the following amino acid sequence: **T-D-T-Q-Y-I-G-R-F-A-P**. This corresponds to the sequence following the second methionine. The size of the molecular markers (M) are given in kDa.

The first methionine in the predicted GluQ-RS protein corresponds to the one located on the bulge of the terminator structure (Figure 4A), which also contains a possible Shine-Dalgarno sequence. However, in related species like *Escherichia fergusonii* that also have the terminator structure, a methionine is not present at that location. In the *S. flexneri* sequence, there is another AUG codon in the same reading frame 27 nucleotides downstream from the one in the terminator. In order to determine which methionine is the start site for translation of the *S. flexneri* GluQ-RS, we constructed a vector that included the intergenic region from the stop codon of the *dksA* gene to the end of *gluQ-rs* cloned into the expression vector pET15c. This allowed expression of C-terminal His-tagged GluQ-RS under T7 promoter control. The protein was partially purified by affinity chromatography as described elsewhere [10], and the sequence of the amino terminus of the GluQ-RS protein was determined to be NH_2_-T-D-T-Q-Y-I-G-R-F-A-P, which corresponds to the amino acid sequence after the second methionine. Therefore, the initiator methionine is not the one indicated in the database, and the protein is 298 amino acids. Surprisingly, there is no obvious Shine-Dalgarno sequence adjacent to the initiator methionine we identified (Figure 5).

### Phenotype of the *S. flexneri gluQ-rs* mutant

**Figure 6:**
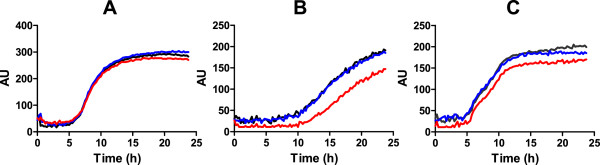
**The*gluQ-rs*mutant is sensitive to growth in high osmolarity.** Biolog phenotypic MicroArrays were used to characterize the growth and metabolism of the *gluQ-rs* mutant and its wild type parent, 2457T. Wild type (black line) transformed with the empty plasmid, 2457T Δ*gluQ-rs::kan* (red line) transformed with the empty plasmid pCM and *S. flexneri* Δ*gluQ-rs::kan* (blue line) transformed with the complementary plasmid pTRCGQ were incubated for 24 h with medium in the corresponding plate for osmolytes (PM9). **A**) sodium chloride 1% **B**) sodium benzoate 20 mM pH 5.2. **C**) sodium nitrate 100 mM. Metabolism was monitored by measuring reduction of the tetrazolium dye in the medium at 15 min intervals and is shown as units.

To determine the role of GluQ-RS in *S. flexneri* growth and virulence, a deletion mutant of the *gluQ-rs* gene was constructed in *S. flexneri* 2457T. The mutant was compared to the wild type by Biolog phenotype MicroArrays (Biolog, Inc., Almeda, CA). The major difference observed for the mutant was impaired metabolism when grown under osmotic stress conditions (Figure 6). The mutant had a longer lag and reduced growth compared to the wild type in the presence of increasing concentrations of potassium chloride, sodium sulfate, sodium formate, sodium benzoate, sodium nitrate and sodium nitrite. The phenotype was complemented with the *gluQ-rs* gene cloned into an expression vector. No differences were observed in the growth or metabolism of these strains when they were incubated in presence of 1% sodium chloride, which was similar to LB (Figure 6 and data not shown).

Because expression of *dksA* is required for *S. flexneri* virulence [27], and growth of *Shigella* in the intracellular environment may induce a stress response, we also measured invasion and plaque formation by the *gluQ-rs* mutant. However, no significant differences were noted (data not shown), suggesting that GluQ-RS is not essential for invasion or intracellular growth of *S. flexneri.*

## Discussion

### Conserved *dksA-gluQ-rs* genomic organization in gammaproteobacteria

GluQ-RS, a paralog of GluRS synthetase, is involved in the formation of GluQ, the nucleoside located at the wobble position of tRNA^Asp^ in bacteria. The protein is present in Firmicutes, Actinobacteria, Cyanobacteria, Alphaproteobacteria, Betaproteobacteria, Gammaproteobacteria and Deltaproteobacteria (Figure 1). From the phylogenetic analysis we distinguished the three subgroups described previously [11] based on the HIGH motif that is present in the class I aminoacyl-tRNA synthetases [2]. As was described previously [11], all GluQ-RS enzymes are characterized by the replacement of a threonine in GluRS enzymes, which is involved in the recognition of the amino acid and the terminal adenosine of the tRNA^Glu^ (Thr_133_ of *Methanocaldococcus jannaschii* GluRS enzyme) by isoleucine, leucine or valine at that position (Ile_47_ of *S. flexneri* GluQ-RS). This substitution is also conserved in all enzymes analyzed here, including those from the Firmicutes group.

The *gluQ-rs* gene is widely distributed in the bacterial domain; however, its genome organization is variable. We observed that only in members of the gammaproteobacteria, namely *Aeromonadales*, *Alteromonadales, Pseudomonadaceae*, *Enterobacteriaceae* and *Vibrionaceae*, the *gluQ-rs* gene is located immediately downstream of the *dksA* gene (Figure 1). A more detailed analysis shows that even within this genomic organization there are differences. In some species of *Pseudomonadaceae*, such as *P. aeruginosa, P. entomophila,* and *P. fluorescens*, we observed the same genomic structure as in *E. coli* or *S. flexneri*, with a distinctive terminator between the genes. In contrast, while the *dksA* gene is also upstream of *gluQ-rs* in some *P. syringae*, there are insertions of an encoded transposase or more than a 400 base pairs separating both genes without a detectable terminator. However, using bioinformatics tools we detected a possible promoter within this region in *P. syringae* (data not shown), indicating that the expression of the *gluQ-rs* gene may be under control of its own promoter, a question that remains to be addressed.

### Stringent response and tRNA modification

Our bioinformatic analysis shows the presence of a transcriptional terminator and lack of a *gluQ-rs*-specific promoter. This is consistent with our results, in which we did not detect any activity from promoters other than those upstream of the *dksA* gene (Figure 3). This unusual arrangement suggests that *gluQ-rs* expression is dependent on *dks*A-regulated conditions. Because DksA is a key member of the stringent response in bacteria and regulates a number of processes in the cell, including its own expression [25,28], the data suggest that there is coordinate regulation of tRNA modification and other DksA targets. Although we could not detect any promoter activity specific for *gluQ-rs* in the growth conditions tested (i.e. altering the pH, presence of glutamate), we cannot discount the possibility that the gene is specifically regulated under some other conditions. The regulon database (http://regulondb.ccg.unam.mx/index.jsp) indicates that the *E. coli gluQ-rs* gene has a recognition site for the σ^24^ subunit of RNA polymerase. From our analysis, this sequence is identical to *S. flexneri*, but there is no experimental evidence of this recognition. Interestingly, when the *gluQ-rs* gene was deleted in *S. flexneri*, the mutant showed impaired growth in the presence of osmolytes (Figure 6). A recent publication demonstrated that σ^24^ and σ^S^ proteins from *Salmonella enterica* serovar Typhi are important for the expression of several genes induced by osmotic stress in this bacterium [29]. Moreover, the expression of the gene encoding σ^24^ in *E. coli* is regulated by the stringent response [30]. The possible role of σ^24^ on the expression of *gluQ-rs* under osmotic stress might be interesting to study.

GluQ-RS is an enzyme responsible for the formation of the GluQ tRNA modification, and two independent groups [10,11] have shown that this enzyme required a high concentration of glutamate to be activated and transferred to the queuosine base present on the tRNA^Asp^. Interestingly, one of the first events to occur when bacteria are subject to high osmolyte stress is an increase in glutamate levels within the cytoplasm [31]. Our observation indicates an important role of the tRNA modification for the growth of *S. flexneri* in the presence of osmolytes (Figure 6). Other tRNA modifications might play a similar role in this stress condition. In *E. coli,* inactivation of the *yfiC* gene, responsible for the modification at the adenosine 37 present on the tRNA^Val^, leads to a high sensitivity to osmotic stress [32].

### Transcription of *gluQ-rs* is regulated by a terminator

The results obtained in the present work show the presence of a terminator and suggested the functionality of this structure (Figure 3 and Figure 4). To our knowledge, there are few examples of bacterial genes that have similar structures. There is a terminator structure upstream of the DNA primase gene, *dnaG,* which also has an unusual Shine Dalgarno sequence [33]. Another example is the *recX* gene in *E. coli,* where readthrough accounts for approximately 10% of its transcription [34].

The two characteristics of *gluQ-rs* described in this work, co-transcription with the upstream gene and the presence of a terminator immediately upstream, allow us to propose that both the transcription and translation process could be regulated in the *gluQ-rs* gene. It has been described, that the presence of terminators upstream of the coding region might be part of a regulatory system such as a riboswitch [35]. Riboswitches for genes involved in queuosine formation have been described, in which the precursor preQ1 is the ligand of the mRNA structure [36]. Using the riboswitch server (ribosw.mbc.nctu.edu.tw [37]), we did not identify any potential riboswitch (data not shown). However we cannot discount that the terminator described here might be part of a regulatory circuit similar to a riboswitch, or that an unidentified protein might bind the terminator structure.

### GluQ modification and codon bias

tRNA modifications present at the anticodon loop might be important for the accuracy of codon reading during the translation processes [13]. Morris et al., 1999 [14] proposed, based on molecular modeling, that the tRNA^AspQ34^ might improve recognition of both GAC and GAU codons, consequently the interaction of the codon GAU with the anticodon of tRNA^AspG34^ could be less efficient. In fact, in *S. flexneri* there are a few genes such as *sitA*, *virF* and *proX* (an inducible gene under osmotic stress) that have a bias toward those codons that favor the modified tRNA. Thus, while there is no obvious loss of plaque formation in the *gluQ-rs* mutant (data not shown), the absence of GluQ-RS may influence the expression of proteins such as SitA that are required for fitness of *Shigella* in the host [38].

## Conclusions

In this work we have shown that the expression of *gluQ-rs*, a gene codifying an enzyme involved in the formation of GluQ present on the tRNA^Asp^, is under the control of the *dksA* promoter. Also, we show the presence of a functional terminator that controls the expression of *gluQ-rs*. Finally, we present data that suggest that the presence of modification of the tRNA^Asp^ is important for survival of the human pathogen *Shigella flexneri* under osmotic stress conditions.

## Methods

### Bacterial growth conditions

The bacterial strains and plasmids used in this study are described in Table 1. *E. coli* strains were maintained on LB-agar (10 g of tryptone per liter, 5 g of yeast extract per liter, 10 g of NaCl per liter and 15 g of agar per liter), *Shigella* strains were maintained on Trypticase Soy Agar plus 0.01% congo red. All strains were stored at −80°C in LB broth plus 20% glycerol. The bacteria were grown in LB broth adjusted to pH 7.4 with 40 mM MOPS (Merck) or M9 minimal media [24]. When necessary, ampicillin was added to a final concentration of 100 μg/ml. Bacterial growth was monitored by optical density at 600 nm (OD_600_).

### Bioinformatics tools to construct the phylogenetic tree

The protein sequences were obtained from the Uniprot database (http://www.uniprot.org/) and then were searched in the GenomeNet (http://www.genome.jp/) to confirm the genomic organization. A selected number of GluQ-RS enzymes were aligned using the MUSCLE algorithm [39] and analyzed using the maximum-likelihood method based on the JTT matrix-based model. The percentage of trees in which the associated proteins clustered together is shown next to the branches. The analysis involved 54 amino acid sequences, including the GluRS proteins from *Methanocaldococcus jannaschii* and *Archaeoglobus fulgidus* as an outgroup. All positions containing gaps and missing data were eliminated. There were a total of 199 positions in the final dataset. Evolutionary analyses were conducted in MEGA5 [21].

### RNA isolation and synthesis of cDNA

Total mRNA was obtained during the growth of *S. flexneri* 2457T using the RNeasy mini kit following the supplier instructions (Qiagen). The purified nucleic acid was treated with RNase- free DNase (Fermentas) and its concentration was estimated by measuring the optical density at 260 nm (OD_260_). Approximately 1 μg of total RNA was subjected to reverse transcription using M-MuLV polymerase (Fermentas) and random primers following the provider’s protocol. The cDNA was amplified using specific PCR primers for each gene of interest (Table 2).

**Table 2:** Primer sequences

**Name**	**Sequence 5**^**′**^**- 3**^**′**^^**a**^	**Reference and characteristics**
opeF	TAAGGAGAAGCAACATGCAAGA	This work. RT-PCR of *dksA* operon from nucleotide +40 to +1477^b^
opeR	ATAGCTCAGCATGACGCATTT	
dksAF	ATGCAAGAAGGGCAAAACCG	This work. RT-PCR of *dksA* gene from nucleotide +54 to +488
dksAR	GCGAATTTCAGCCAGCGTTT	
interF	AGTGGAAGACGAAGATTTCG	This work, RT-PCR of intergenic region from nucleotide +368 to +863
interR	TCCTTGTTCATGTAACCAGG	
gQRSF	TTCAAAGAGATGACAGACACACAG	This work, RT-PCR of *gluQ-rs* gene from nucleotide +567 to +1074
gQRSR	CACGGCGATGAATGATAAAATC	
rrsHF	CCTACGGGAGGCAGCAG	[40] RT-PCR of ribosomal RNA 16S
rrsHR	CCCCCGTCAATTCCTTTGAGTTT	
pcnBR	GATGGAGCCGAAAATGTTGT	Reverse of *pcnB* gene from nucleotide +1993
PdksAF	**GGATCC**AAGCGAAGTAAAATACGG	*Bam*HI site, from nucleotide −506
PdksARST	**AAGCTT**GTGATGGAACGGCTGTAAT	*Hin*dIII site, to nucleotide +527
PdksARCT	**AAGCTT**CTGTGTGTCTGTCATCTCTTTG	*Hin*dIII site, to nucleotide +590
PgluQF	**GGATCC**AAGAAGGGCAAAACCGTA	*Bam*HI site, from nucleotide +58
TERGQ2	CCTTATTTTTTGTTCAAAGAGATGACAGACACACAGA	Recognition from nucleotide +555
TERMGQ3	ATAAGGCGGGAGCATAACGGAGGAGTGGTAAAC	Recognition from nucleotide +560, underline sequence are nucleotides changed
M13R	GCGGATAACAATTTCACACAGG	Recognition site in pTZ57R/T
ATGGQRSF	**GGATCC**GTAATTACAGCCGTTCCATC	*Bam*HI site, from nucleotide +507. Underline nucleotides correspond to the stop codon of *dksA*
ATGGQRSR	**CTCGAG**GCATGACGCATTTGAGAATG	*Xho*I site, to nucleotide +1469
virFF	AGCTCAGGCAATGAAACTTTGAC	[41]
virFR	TGGGCTTGATATTCCGATAAGTC	

^a^Nucleotides in bold are indicated restriction site.

^b^Fragments cloned are indicated based on the transcription start of *dksA* identified by [25].

### Construction of transcriptional fusions

Transcriptional fusions were constructed to study the expression control of *gluQ-rs*. Fragments of the *dksAgluQ-rs* region were fused to *lacZ* in the vector pQF50 by using the *Bam*HI and *Hin*dIII restriction sites [23]. Each fragment was amplified from *S. flexneri* genomic DNA using the indicated primers (Tables 1 and 2) with the High Fidelity PCR Enzyme Mix polymerase (Fermentas) and cloned into pQF50 (Table 1). Once the sequence of each clone was confirmed, the recombinant plasmid was introduced into *S. flexneri* 2457T by electroporation. The nomenclature of the recombinants plasmids is: P for promoter of the *dksA* gene, D for the *dksA* gene and T for a terminator structure.

### β-galactosidase activity

*S. flexneri* transformed with the corresponding constructs were cultured overnight in LB, a 1:50 dilution was inoculated into 10 ml culture of LB pH 7.4 and grown to an OD_600_ of 0.5. Aliquots of 0.5 ml of each strain containing the clone or the empty vector were assayed for β-galactosidase activity according to Miller [42]. The data were analyzed using the software GraphPad Prism V5.01.

### Site directed mutagenesis

A possible transcription terminator between *dksA* and *gluQ-rs* was identified using the program Mfold [26]. Site directed mutagenesis by overlap PCR was performed to disrupt the predicted terminator [43]. Using the fragment VCPDT cloned in the vector pTZ57R/T as template, was amplified a 1,072 bp fragment, which include the mutation, using the primers PdksAF and TERMGQ3, while a second fragment of 162 bp overlapping the mutated region, was obtained with primers TERGQ2 and M13R (Table 2). Both fragments (1,072 bp and 162 bp) were digested with *Dpn*I, purified and mixed at equimolar quantities to carry out a PCR reaction using the 5^′^ and 3^′^ ends primers (PdksAF and PdksARCT). The 1,110 bp amplified fragment was cloned in the vector pTZ57R/T and sequenced to verify the mutation. This plasmid was digested with *Bam*HI and *Hin*dIII and the fragment subcloned in to the vector pQF50.

### Determination of first methionine of GluQ-RS

In order to establish which is the first AUG codon of *gluQ-rs,* the recombinant plasmid pATGGQRS was constructed. A PCR reaction was performed using the primers ATGGQRSF and ATGGQRSR (Table 2) and genomic DNA from *S. flexneri*. The amplified fragment, containing the *Bam*HI site, stop codon of *dksA*, the intergenic region with the terminator, the *gluQ-rs* reading frame without its stop codon and the *Xho*I site was cloned into pET15c, a modified version of pET15b, which was constructed by inserting the 290 bp *Xba*I and *Blp*I fragment of pET20b containing the polylinker into pET15b. This construct allowed the synthesis of a C-terminal histidine tagged protein under the transcription control of the T7 promoter. The construct was transformed in BL21(DE3) strain and the His-tagged protein was partially purified by affinity chromatography as described previously [10]. The eluted protein was transferred to a PVDF membrane and stained with Coomassie blue. The predominant band of the expected size (34.6 kDa) was sequenced at the Protein Core Facility of the Institute for Cellular and Molecular Biology, University of Texas at Austin.

### Construction of the plasmid for complementation of the *gluQ-rs* mutation

This plasmid was constructed from the pATGGQRS plasmid in which the T7 promoter was removed by digestion with *Bgl*II and *Nco*I enzymes and replaced by the TRC promoter obtained from pTRC99a plasmid by amplification and digestion with *Bam*HI and *Nco*I to obtain the pTRCGQ plasmid. The empty plasmid (pCM) was constructed by incorporating the TRC promoter into the pET15c plasmid.

### Inactivation of *gluQ-rs* gene in *S. flexneri*

Deletion of *gluQ-rs* was carried out using the λ red recombinase method [44] with the following modifications. *S. flexneri* 2457T carrying pKD46 and prepared as described elsewhere [44] was transformed with a purified PCR fragment amplified from the *E. coli* Δ*gluQ-rs::kan* mutant strain using primers dksAF and pcnBR (Table 2), increasing the homologous DNA region to more than 450 bp at each side. The mutant was isolated following overnight growth at 37°C on LB-agar containing kanamycin (50 μg/ml). The deletion was confirmed by PCR using the same pair of primers (dksAF-pcnBR) and using each primer together with an internal primer as described previously [44]. The presence of the *S. flexneri* virulence plasmid was also confirmed by PCR amplification of the *virF* gene using primers virFF and virFR (Table 2).

### Effect of the absence of *gluQ-rs* gene in *S. flexneri* metabolism

The effect of the deletion of the *gluQ-rs* gene on the metabolism of *S. flexneri* was analyzed by Biolog phenotype MicroArrays following the manufacturer’s instructions (Biolog, Inc., Almeda, CA). Strains were grown at 30°C overnight and 5 ml of LB was inoculated with a 1:100 dilution and grown at 37°C to reach an OD_650nm_ of 0.5. The cells were then washed and resuspended to 2.5 x 10^7^ cfu/ml and diluted 200 fold in to a solution of IF-10a medium (Biolog). Each well was inoculated with 1.2 x 10^4^ cfu (0.1 ml per well) into the corresponding plates and incubated for 24 hrs at 37°C. The metabolism was recorded and analyzed by the Omnilog software (V 1.20.02) (Biolog, Inc., Almeda, CA).

## Abbreviations

GluQ-RS: Glutamyl queuosine-tRNA^Asp^ synthetase and its codifying gene; tRNA^Asp^: Transfer ribonucleic acid codifying aspartic acid; GluQ: Glutamyl-queuosine; aaRS: Aminoacyl–tRNA synthetase; GluRS: Glutamyl-tRNA synthetase; Q: Queuosine; SPI-1: Pathogenicity islands of Salmonella; *miaA*: tRNA Δ(2)-isopentenylpyrophosphate transferase; *tgt*: tRNA guanine transglycosylase; RT-PCR: Reverse transcription-polymerase chain reaction; LB: Luria Bertani; MOPS: 3-(N-morpholino)propanesulfonic acid; M-MuLV: Moloney Murine Leukemia Virus Reverse Transcriptase; PVDF: Polyvinyl difluoride.

## Competing interests

The authors declare that they have no competing interests.

## Authors’ contributions

VCC: performed cloning, the enzymatic assay, data analysis. VPT: performed *ex vivo* assays of the wild type and mutant strains of *Shigella* and the enzymatic activity under different growth conditions. CM: performed the RT-PCR of the mRNA isolated from *Shigella flexneri*. CRF: performed the Biolog assay in collaboration with JCS. JCS: Analysis of the data, experimental design, N terminal determination of GluQ-RS. SMP and JCS: analyzed the results and wrote the paper. All authors contributed to the editing and approved the final paper.

## References

[B1] Ibba M Söll D Aminoacyl-tRNA synthesis Annu Rev Biochem 2000 69 617 650 10.1146/annurev.biochem.69.1.617 10966471

[B2] Eriani G Delarue M Poch O Gangloff J Moras D Partition of tRNA synthetases into two classes based on mutually exclusive sets of sequence motifs Nature 1990 347 203 206 10.1038/347203a0 2203971

[B3] Woese CR Olsen GJ Ibba M Söll D Aminoacyl-tRNA synthetases, the genetic code, and the evolutionary process Microbiol Mol Biol Rev 2000 64 202 236 10.1128/MMBR.64.1.202-236.2000 10704480 PMC98992

[B4] Skouloubris S de Pouplana LR de Reuse H Hendrickson H A noncognate aminoacyl-tRNA synthetase that may resolve a missing link in protein evolution Proc Natl Acad Sci USA 2003 100 11297 11302 10.1073/pnas.1932482100 13679580 PMC208751

[B5] Salazar JC Ahel I Orellana O Tumbula-Hansen D Krieger R Daniels L Söll D Coevolution of an aminoacyl-tRNA synthetase with its tRNA substrates Proc Natl Acad Sci USA 2003 100 13863 13868 10.1073/pnas.1936123100 14615592 PMC283512

[B6] Schimmel P Ripmaster T Modular design of components of the operational RNA code for alanine in evolution Trends Biochem Sci 1995 20 333 334 10.1016/S0968-0004(00)89067-2 7482695

[B7] Sissler M Delorme C Bond J Ehrlich SD Renault P Francklyn C An aminoacyl-tRNA synthetase paralog with a catalytic role in histidine biosynthesis Proc Natl Acad Sci USA 1999 96 8985 8990 10.1073/pnas.96.16.8985 10430882 PMC17719

[B8] Navarre WW Zou SB Roy H Xie JL Savchenko A Singer A Edvokimova E Prost LR Kumar R Ibba M Fang FC PoxA, YjeK, and elongation factor P coordinately modulate virulence and drug resistance in Salmonella enterica Mol Cell 2010 39 209 221 10.1016/j.molcel.2010.06.021 20670890 PMC2913146

[B9] Bearson SM Bearson BL Brunelle BW Sharma VK Lee IS A mutation in the poxA gene of Salmonella enterica serovar Typhimurium alters protein production, elevates susceptibility to environmental challenges, and decreases swine colonization Foodborne Pathog Dis 2011 8 725 732 10.1089/fpd.2010.0796 21348575

[B10] Salazar JC Ambrogelly A Crain PF McCloskey JA Söll D A truncated aminoacyl–tRNA synthetase modifies RNA Proc Natl Acad Sci USA 2004 101 7536 7541 10.1073/pnas.0401982101 15096612 PMC419641

[B11] Dubois DY Blaise M Becker HD Campanacci V Keith G Giegé R Cambillau C Lapointe J Kern D An aminoacyl-tRNA synthetase-like protein encoded by the Escherichia coli yadB gene glutamylates specifically tRNAAsp Proc Natl Acad Sci USA 2004 101 7530 7535 10.1073/pnas.0401634101 15096594 PMC419640

[B12] Iwata-Reuyl D Biosynthesis of the 7-deazaguanosine hypermodified nucleosides of transfer RNA Bioorg Chem 2003 31 24 43 10.1016/S0045-2068(02)00513-8 12697167

[B13] Gustilo EM Vendeix FA Agris PF tRNA’s modifications bring order to gene expression Curr Opin Microbiol 2008 11 134 140 10.1016/j.mib.2008.02.003 18378185 PMC2408636

[B14] Morris RC Brown KG Elliott MS The effect of queuosine on tRNA structure and function J Biomol Struct Dyn 1999 4 757 77414 10217448 10.1080/07391102.1999.10508291

[B15] Harada F Nishimura S Possible anticodon sequences of tRNAHis, tRNAAsnand tRNAAspfromEscherichia coliB. Universal presence of nucleoside Q in the first position of the anticodons of these transfer ribonucleic acids Biochem 1972 11 301 308 10.1021/bi00752a024 4550561

[B16] Durand JM Okada N Tobe T Watarai M Fukuda I Suzuki T Nakata N Komatsu K Yoshikawa M Sasakawa C vacC, a virulence-associated chromosomal locus of Shigella flexneri, is homologous to tgt, a gene encoding tRNA-guanine transglycosylase (Tgt) of Escherichia coli K-12 J Bacteriol 1994 176 4627 4634 8045893 10.1128/jb.176.15.4627-4634.1994 PMC196283

[B17] Durand JM Björk GR Kuwae A Yoshikawa M Sasakawa C The modified nucleoside 2-methylthio-N6-isopentenyladenosine in tRNA of Shigella flexneri is required for expression of virulence genes J Bacteriol 1997 179 5777 5782 9294434 10.1128/jb.179.18.5777-5782.1997 PMC179466

[B18] Urbonaviĉius J Qian Q Durand JM Hagervall TG Björk GR Improvement of reading frame maintenance is a common function for several tRNA modifications EMBO J 2001 20 4863 4873 10.1093/emboj/20.17.4863 11532950 PMC125605

[B19] Szklarczyk D Franceschini A Kuhn M Simonovic M Roth A Minguez P Doerks T Stark M Muller J Bork P Jensen LJ Mering C The STRING database in 2011: functional interaction networks of proteins, globally integrated and scored Nucleic Acids Res 2011 39 D561 D568 10.1093/nar/gkq973 21045058 PMC3013807

[B20] Kanehisa M Linking databases and organisms: GenomeNet resources in Japan TIBS 1997 22 442 444 9397687 10.1016/s0968-0004(97)01130-4

[B21] Tamura K Peterson D Peterson N Stecher G Nei M Kumar S MEGA5: molecular evolutionary genetics analysis using maximum likelihood, evolutionary distance, and maximum parsimony methods Mol Biol Evol 2011 28 2731 2739 10.1093/molbev/msr121 21546353 PMC3203626

[B22] Kang PJ Craig EA Identification and characterization of a new Escherichia coli gene that is a dosage-dependent suppressor of a dnaK deletion mutation J Bacteriol 1990 172 2055 2064 2180916 10.1128/jb.172.4.2055-2064.1990 PMC208704

[B23] Farinha MA Kropinski AM Construction of broad-host-range plasmid vectors for easy visible selection and analysis of promoters J Bacteriol 1990 172 3496 3499 2111810 10.1128/jb.172.6.3496-3499.1990 PMC209165

[B24] Sambrook J Fritsch EF Maniatis T Molecular Cloning: A Laboratory Manual 1989 2 USA: Cold Spring Harbor Laboratory Press

[B25] Chandrangsu P Lemke JJ Gourse RL The dksA promoter is negatively feedback regulated by DksA and ppGpp Mol Microbiol 2011 80 1337 1348 10.1111/j.1365-2958.2011.07649.x 21496125 PMC3103630

[B26] Zuker M Mfold web server for nucleic acid folding and hybridization prediction Nucleic Acids Res 2003 31 3406 3415 10.1093/nar/gkg595 12824337 PMC169194

[B27] Mogull SA Runyen-Janecky LJ Hong M Payne SM dksA is required for intercellular spread of Shigella flexneri via an RpoS-independent mechanism Infect Immun 2001 69 5742 5751 10.1128/IAI.69.9.5742-5751.2001 11500451 PMC98691

[B28] Sharma UK Chatterji D Transcriptional switching in Escherichia coli during stress and starvation by modulation of sigma activity FEMS Microbiol Rev 2010 34 646 657 20491934 10.1111/j.1574-6976.2010.00223.x

[B29] Du H Wang M Luo Z Ni B Wang F Meng Y Xu S Huang X Coregulation of gene expression by sigma factors RpoE and RpoS in Salmonella enterica serovar Typhi during hyperosmotic stress Curr Microbiol 2011 62 1483 1489 10.1007/s00284-011-9890-8 21311887

[B30] Durfee T Hansen A-M Zhi H Blattner FR Jin DJ Transcription profiling of the stringent response in Escherichia coli J Bacteriol 2008 190 1084 1096 10.1128/JB.01092-07 18039766 PMC2223561

[B31] Booth IR Higgins CF Enteric bacteria and osmotic stress: intracellular potassium glutamate as a secondary signal of osmotic stress? FEMS Microbiol Rev 1990 6 239 246 1974769 10.1111/j.1574-6968.1990.tb04097.x

[B32] Golovina AY Sergiev PV Golovin AV Serebryakova MV Demina I Govoru VM Dontsova OA The yfiC gene of E. coli encodes an adenine-N6 methyltransferase that specifically modifies A37 of tRNA1Val (cmo5UAC) RNA 2009 15 1134 1141 10.1261/rna.1494409 19383770 PMC2685529

[B33] Smiley BL Lupski JR Svec PS McMacken R Godson GN Sequences of the Escherichia coli dnaG primase gene and regulation of its expression Proc Natl Acad Sci USA 1982 79 4550 4554 10.1073/pnas.79.15.4550 6750604 PMC346712

[B34] Pagès V Koffel-Schwartz N Fuchs RPP recX, a new SOS gene that is co-transcribed with the recA gene in Escherichia coli DNA Repair 2003 2 273 284 10.1016/S1568-7864(02)00217-3 12547390

[B35] Garst AD Edwards AL Batey RT Riboswitches: structures and mechanisms Cold Spring Harbor Perspect Biol 2011 3 a003533 10.1101/cshperspect.a003533 PMC3098680 20943759

[B36] Roth A Winkler WC Regulski EE Lee BWK Lim J Jona I Barrick JE Ritwik A Kim JN Welz R Iwata-Reuyl D Breaker RR A riboswitch selective for the queuosine precursor preQ1 contains an unusually small aptamer domain Nat Struct Mol Biol 2007 14 308 317 10.1038/nsmb1224 17384645

[B37] Chang TH Huang HD Wu LC Yeh CT Liu BJ Horng JT Computational identification of riboswitches based on RNA conserved functional sequences and conformations RNA 2009 15 1426 1430 10.1261/rna.1623809 19460868 PMC2704089

[B38] Fisher CR Davies NM Wyckoff EE Feng Z Oaks EV Payne SM Genetics and virulence association of the Shigella flexneri sit iron transport system Infect Immun 2009 77 1992 1999 10.1128/IAI.00064-09 19289511 PMC2681762

[B39] Edgar RC MUSCLE: multiple sequence alignment with high accuracy and high throughput Nucleic Acids Res 2004 32 1792 1797 10.1093/nar/gkh340 15034147 PMC390337

[B40] Yu Z Morrison M Comparisons of different hypervariable regions of rrs genes for use if fingerprinting of microbial communities by PCR-Denaturing Gel Electrophoresis Appl Environ Microbiol 2004 70 4800 4806 10.1128/AEM.70.8.4800-4806.2004 15294817 PMC492348

[B41] Vidal M Kruger E Durán C Lagos R Levine M Prado V Toro C Vidal R Single multiplex PCR assay to identify simultaneously the six categories of diarrheagenic Escherichia coli associated with enteric infections J Clin Microbiol 2005 43 5362 5365 10.1128/JCM.43.10.5362-5365.2005 16208019 PMC1248459

[B42] Miller J Experiments in Molecular Genetics 1972 NY: Cold Spring Harbor Laboratory 352 355

[B43] Ho SN Hunt HD Horton RM Pullen JK Pease LR Site-directed mutagenesis by overlap extension using the polymerase chain reaction Gene 1989 77 51 59 10.1016/0378-1119(89)90358-2 2744487

[B44] Datsenko KA Wanner BL One-step inactivation of chromosomal genes in Escherichia coli K-12 using PCR products Proc Natl Acad Sci USA 2000 97 6640 6645 10.1073/pnas.120163297 10829079 PMC18686

